# Open-source implementation of polarisation-resolved single-shot differential phase contrast microscopy (*pDPC*) on a modular *openFrame*-based microscope

**DOI:** 10.1016/j.ohx.2024.e00622

**Published:** 2024-12-27

**Authors:** Huihui Liu, Sunil Kumar, Edwin Garcia, William Flanagan, Jonathan Lightley, Christopher Dunsby, Paul M.W. French

**Affiliations:** a LIGHT Community, Physics Department, Imperial College London SW7 2AZ, UK; b The Francis Crick Institute, 1 Midland Road, London NW1 1AT, UK; c Department of Surgery & Cancer, Imperial College London SW7 2AZ, UK

**Keywords:** Quantitative phase imaging, Differential phase contrast, Polarisation, Microscopy

## Abstract

We recently demonstrated polarisation differential phase contrast microscopy (*pDPC*) as a robust, low-cost single-shot implementation of (semi)quantitative phase imaging based on differential phase microscopy. *pDPC* utilises a polarisation-sensitive camera to simultaneously acquire four obliquely transilluminated images from which phase images mapping spatial variation of optical path difference can be calculated. *pDPC* microscopy can be implemented on existing or bespoke microscopes and can utilise radiation at a wide range of visible to near infrared wavelengths and so is straightforward to integrate with fluorescence microscopy. Here we present a low-cost open-source *pDPC* module that is designed for use with the modular open-source microscope stand “*openFrame*”. With improved hardware and software, this new *pDPC* implementation provides a real-time readout of phase across a field of view that facilitates optimisation of system alignment. We also provide protocols for background subtraction and correction of crosstalk.

## Graphical abstract

**Figure:**
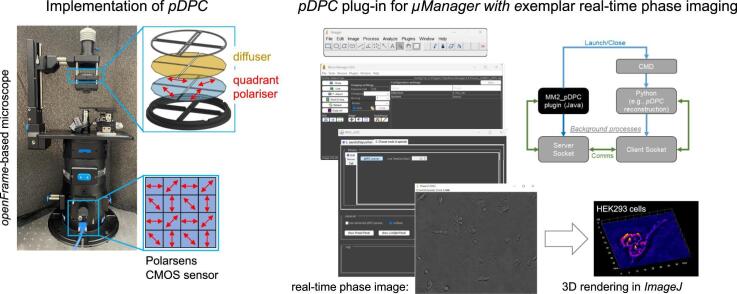

## 1 Specifications table

**Table:** 

Hardware name	*openFrame* polarisation differential phase contrast (*pDPC*) microscope
Subject area	• Biological sciences (e.g., microbiology and biochemistry) • Educational tools and open source alternatives to existing infrastructure
Hardware type	• Imaging tools
Closest commercial analog	Phasefocus Livecyte; Tomocube HT; Telight Q-Phase;
Open source license	CERN Open Hardware License Version 2 Permissive (hardware) BSD 3-Clause license (software)
Cost of hardware	∼£2.5 k excluding brightfield illumination setup (additional ∼£3k on *openFrame*-based microscope) Cost estimates exclude VAT
Source file repository	10.5281/zenodo.14536403 (hardware) 10.5281/zenodo.14536077 (software)

## 2 Hardware in context

Quantitative phase imaging (QPI) maps the variation of optical path difference (OPD) across a field of view (FOV), providing information about the 3D shape of transparent samples. For cell biology it can provide a label-free means to characterize cell morphology and to segment cells for further analysis, including tracking live cell trajectories, sorting cell types, classifying phenotypes, and imaging cell dynamics, e.g., for microbiology, neuroscience, and pathology. As reviewed, for example in Park et al. [3], QPI can be realised in many ways, including explicit interferometric techniques such as phase stepping interferometry, intensity-based computational techniques such as transport of intensity, differential phase contrast (DPC), (Fourier) ptychography, and wavefront sensing techniques, e.g., using Shack Hartmann sensors. Most QPI techniques require specialist components, e.g., interferometer set-ups, objective lenses with phase rings, Nomarski prisms, wavefront sensors, and/or programmable illumination schemes, and most require multiple sequentially acquired images from which to calculate the variation in OPD across the FOV. These requirements increase cost, complexity and/or data acquisition times of QPI instrumentation.

DPC [4] is a non-interferometric QPI technique that maps the OPD variation across a sample by acquiring multiple images under specific modulated light patterns at the source plane of the microscope (i.e., the back focal plane (BFP) of the condenser lens) or its conjugate (e.g., the objective lens pupil plane). In conventional Köhler transillumination, DPC can be implemented by sequentially introducing opaque masks in this BFP source (or conjugate) plane [5], [6]. Alternative DPC implementations have utilized a programmable light-emitting-diode (LED) array [7] without a condenser lens to replace the conventional Köhler illumination set-up. DPC techniques assume weakly scattering samples and provide phase (OPD) images that lack the DC spatial frequency component; hence we describe them as semiquantitative because they do not fully reconstruct the phase profiles of all spatial structures. However, they typically perform well with biological samples such as cultured cells.

DPC requires at least two images acquired under different conditions (typically corresponding to different intensity modulated patterns at the condenser BFP or its conjugate plane). When acquired sequentially, this decreases the QPI time resolution, and the phase (OPD) reconstruction can be prone to motion artefacts. To address this, some single-shot DPC techniques have been proposed, e.g., utilizing a colour camera and color-coded modulation patterns at the condenser BFP [8], [9], [10]. In particular, Philips et al. [8] inserted a quadrant colour filter mask at the condenser BFP in a conventional Köhler transillumination arm and Lee et al. [9] utilized a RGB LED array instead of a single-color LED array to realize condenser-less single-shot DPC. Fan et al. [10] subsequently used a liquid crystal display (LCD) to introduce more complex modulation patterns for an isotropic frequency response.

Colour-multiplexed single-shot DPC techniques typically occupy a wide spectral range, which makes them difficult to integrate with fluorescence imaging. To address this issue, we developed a polarisation-resolved single-shot DPC (*pDPC*) technique [1], which utilizes a polarisation-resolving camera (“PolCam”, based on the Sony Polarsens™ sensor [11]) and incorporates a quadrant polariser (QP) mask at the condenser BFP in a conventional Köhler setup. *pDPC* can be implemented with (quasi-monochromatic) radiation at any wavelength within the sensitivity range of the PolCam and enables QPI to be implemented with existing transillumination light sources (typically with an additional narrowband filter) and to be easily combined with fluorescence microscopy to provide complementary information, e.g., to enable label-free segmentation and tracking of cells for analysis of fluorescence image data.

While we note that there are other single-shot QPI approaches, these are typically based on digital (off-axis) holography, e.g., [12], or phase stepping holography, e.g. [13], requiring interferometers, or shearing/differential interferometric measurements of phase gradients [14], [15], [16], typically requiring modification of the imaging path – often with specially fabricated components – and facing the challenge of integrating the acquired phase gradient data to obtain quantitative phase profiles. In contrast, *pDPC* requires no modification of the imaging path beyond replacing the camera with a PolCam, and the modification of the transillumination light path is straightforward, not requiring any programmable illumination.

In this paper, we present a low-cost implementation of condenser-based *pDPC* on a modular open-source *openFrame*-based microscope [2] that is part of the range of open-source instruments [17] that we are developing to widen access to research microscopy techniques in lower-resourced settings. The basic modular *openFrame* microscope stand is shared as open-source hardware for light microscopy that enables users to assemble a wide range of instruments with no requirements to use proprietary software or hardware components, although it is designed to be easily integrated with a wide range of commercial optomechanical components and microscopy sub-systems or self-built components. *pDPC* can be easily implemented on an *openFrame*-based microscope and it is straightforward to combine it with other imaging modalities, including fluorescence microscopy. We note that the open-source *pDPC* module here was specifically designed for the *openFrame* ecosystem but could be integrated with other microscopes, including home-built or alternative open-source/modular platforms, e.g., [18], [19], [20], [21], that have the space, scale and mechanical strength to support the transillumination pillar.

The *pDPC* image data reported in this paper was acquired using *µManager* [22] and processed by a customized *µManager* plug-in (“MM2_pDPC” [23]) that calculates the phase (OPD) image and provides a live preview of it within the *µManager* graphical user interface (GUI). As illustrated in Supplementary Fig. S1, this plug-in deinterleaves the four polarisation sub-image channels from the raw PolCam image data acquired by *µManager* and, using an analogous approach to that in Tian and Waller [7], the phase component of the optical transmission function of the sample is calculated from these sub-images. The plug-in can also use a pre-determined (“calibration”) system response matrix to correct any unbalanced illumination and crosstalk between the different polarisation channels.

As discussed in Kalita et al. [1], *pDPC* generally offers comparable performance to other differential phase contrast microscopy techniques implemented with similar illumination and imagine numerical aperture optical systems, with the advantages that *pDPC* is single-shot, wavelength agnostic (within the sensitivity and polarisation extinction range of the Polarsens™ sensor [11]) and straightforward to implement at low cost. As discussed in Tian and Waller [7], differential phase contrast microscopy techniques generally suffer from limitations associated with the weak object assumption, are missing low spatial frequency components, and require the illumination numerical aperture (NA) to be not less than the imaging NA, which can particularly limit resolution in condenser-based implementations. Further, any deviation from the illumination source function used in the analysis can also impact the calculated phase. Accordingly, we describe *pDPC* as a semiquantitative phase imaging technique as we do not expect to recover the absolute phase profiles of a sample but rather to capture its morphology, which is sufficient for applications such as label-free segmentation and tracking of cells. Supplementary Fig. S3 presents co-registered *pDPC* phase and fluorescence images of live (DU145) cells acquired on an *openFrame*-based microscope; we can use segmentation based on such *pDPC* phase images to track cells over time without continuous exposure to fluorescence excitation light.

In *pDPC* implemented with condenser-based illumination the illumination NA is practically limited to ∼0.6. This is not sufficient for use with most 40x objective lenses (typically 0.75NA), and we routinely use *pDPC* with 20x, 10x and 4x magnification objectives. Supplementary Fig. S4 shows *pDPC* phase images of mycobacteria (M. smegmatis) acquired with a 0.4 NA 20x magnification objective lens. It will be seen that these mycobacteria phase images present a width comparable to the diffraction-limited resolution for the 0.4 NA objective lens at the wavelength used. For lower magnification objectives, the spatial resolution of *pDPC* is more likely to be limited by the camera pixel size rather than the NA of the objective lens, noting that the *pDPC* uses 2 x 2 arrays of 3.45 µm PolCam pixels as single resolution elements. This may be a disadvantage of *pDPC* compared to other DPC techniques that can utilize smaller camera pixel sizes.

The phase discrimination of QPI microscopes may be estimated as the standard deviation of the phase of the background, e.g. [24]. We expect *pDPC* to provide similar phase resolution to other DPC microscopes using similar optical components. Supplementary Fig. S6 shows how this varies with the camera pixel value in our *pDPC* microscope, decreasing with increasing signal as expected. Also shown is the change in standard deviation of the calculated phase value corresponding to the depth of the mycobacteria as a function of increasing camera signal.

## 3 Hardware description

The implementation of *pDPC* that we present here includes both the open-source hardware for *openFrame*-based microscopes and a new *µManager* plug-in that provides an all-in-one image data processing capability including a real-time display of phase (OPD) image.

**Fig. 1:**
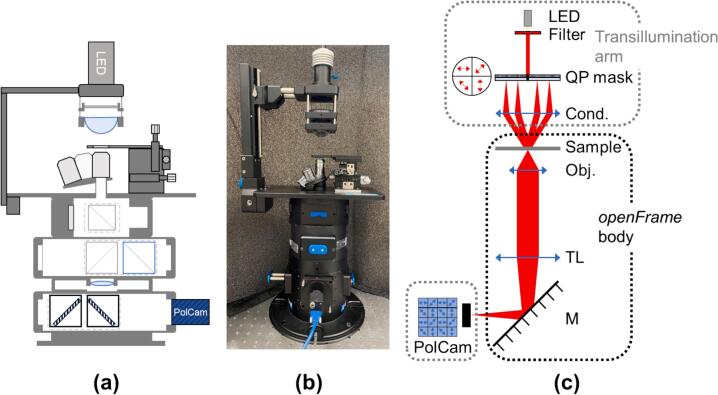
(a) Schematic, (b) photograph, and (c) optical path of the openFrame microscope stand configured for *pDPC*.

The specific *pDPC* hardware is presented in Fig. 1(a, b) with Fig. 1(c) showing the optical configuration. It includes a standard *openFrame* microscope stand configured for brightfield Köhler transillumination imaging utilising a low-cost white LED and a condenser lens that permits a maximum illumination numerical aperture (NA) of 0.55 after the QP mask. *pDPC* is implemented by mounting a QP mask at the condenser BFP using a 2-inch (Thorlabs) optomechanical cage system.

The QP mask utilises low-cost polariser quadrants cut from polarising polymer film (Edmund Optics, #29-494). A diffusing layer cut from standard tracing paper is placed above each polariser to create an apparent source plane that matches the source function model in the *pDPC* phase reconstruction process [7].

On the detection side, *pDPC* simply requires a PolCam mounted as for standard brightfield imaging.

The cost to add *pDPC* to the brightfield microscope is <£3000 including the PolCam, optomechanical/cage components, the QP assembly (which is 3D-printed), and some customized tools that are used to “calibrate” (i.e., measure the system response matrix of) the *pDPC* system. All CAD files for the customized (3D-printed &/ CNC) parts were generated and are publicly shared via the “*Onshape*” open CAD platform and are available at [25]. This *Onshape* repository has been configured to accommodate further customization, details for which can be found at [25].

We note that the components used were selected for wide availability, but they could be replaced by other similar components. For example, polarisation-resolving cameras based on the Polarsens™ sensor are now available from more than 15 manufacturers (see [11]) and the Thorlabs cage system could be replaced by other optomechanical mounting systems.

## 4 Design files summary

There are three types of CAD files listed in Table 1: SLDPRT, STEP, and PDF drawings. SLDPRT and STEP files are provided for 3D printing parts, while PDF drawings are provided for CNC.

**Table 1:** Design files for 3D printing, CNC, and software.

Design file name	File type	Open source license	Location of the file
**QPM.zip**			
QPM_holder_base	SLDPRT & STEP	CERN Open Hardware License Version 2 Permissive	10.5281/zenodo.14536403
QPM_holder_cover	SLDPRT & STEP	CERN Open Hardware License Version 2 Permissive	10.5281/zenodo.14536403
QPM_quadrant_cutmodel	(A) SLDPRT & STEP	CERN Open Hardware License Version 2 Permissive	10.5281/zenodo.14536403
	(B) PDF drawing	CERN Open Hardware License Version 2 Permissive	10.5281/zenodo.14536403

**CaliMask.zip**			
CaliMask_source	SLDPRT & STEP	CERN Open Hardware License Version 2 Permissive	10.5281/zenodo.14536403
CaliMask_pupil	SLDPRT & STEP	CERN Open Hardware License Version 2 Permissive	10.5281/zenodo.14536403
CaliMask_pupil_adapter	PDF drawing	CERN Open Hardware License Version 2 Permissive	10.5281/zenodo.14536403

**Software.zip**			
MM2_pDPC	JAR	BSD 3-Clause license	10.5281/zenodo.14536077
MM2_pDPC_src	ZIP of source codes	BSD 3-Clause license	10.5281/zenodo.14536077

QPM.zip contains the CAD files of all components used to fabricate the QP mask. QPM_holder_base and QPM_holder_cover are supporting structures of the QP mask and are made by 3D printing, while QPM_quadrant_cut model is an auxiliary part to assist with the fabrication process and can be made by either 3D printing or CNC.

CaliMask.zip contains the CAD files of all components which can be used to determine the *pDPC* system response. This can be undertaken using a 3D-printed mask that blocks three of the four quadrants in the source plane (CaliMask_source) or using a different mask positioned near the pupil plane of the objective lens (CaliMask_pupil) that restricts the light path into a single quadrant. CaliMask_pupil_adapter is an auxiliary part used to mount CaliMask_pupil.

Software.zip contains the MM2_pDPC JAR file, which can be directly used to install the *µManager* plugin for *pDPC* reconstruction, and the MM2_pDPC_src ZIP file containing all source files used to compile the JAR file.

## 5 Bill of materials summary

Note that all prices in Table 2 are exc. VAT and all contents were updated on 06/06/2024 unless specifically stated in the table.

**Table 2:** Bill of materials.

Designator	Component	Number	Cost per unit −currency	Total cost − currency	Source of materials	Material type
**1. Basic *openFrame* transillumination module for brightfield imaging**						
1-1	Cairn Rack and pinion tilt-back pillar, P1136/000/000	1	£1,650.00 (updated 25/07/2023)	£1,650.00	Cairn Research Ltd https://cairn-research.co.uk/	Metal
1-2	Thorlabs Ø12.7 mm post, TR75/M	2	£4.92	£9.84	https://www.thorlabs.com/thorproduct.cfm?partnumber=TR75/M	Metal
1-3	Cairn MonoLED adapter plate, P1136/LED/000	1	£250.00 (updated 25/07/2023)	£250.00	Cairn Research Ltd https://cairn-research.co.uk/	Metal
1-4	Cairn White MonoLED, P1125/000/WHT	1	£1,040.00 (updated 25/07/2023)	£1,040.00	Cairn Research Ltd https://cairn-research.co.uk/	Non-specific

**2. *pDPC* condenser-based transillumination module**						
2-1	Semrock BrightLine® 628/40 nm single-band bandpass filter, FF02-628/40-25	1	$375.00	$375.00	https://www.idex-hs.com/store/product-detail/ff02_628_40_25/fl-004435	Glass
2-2	Thorlabs Ø6 mm cage rods (4 Pack), ER3-P4	1	£21.70	£21.70	https://www.thorlabs.com/thorproduct.cfm?partnumber=ER3-P4	Metal
2-3	Thorlabs SM2 retaining ring, SM2RR	1	£6.10	£6.10	https://www.thorlabs.com/thorproduct.cfm?partnumber=SM2RR	Metal
2-4	QP mask	1	See QP mask section below			Non-specific
2-5	Thorlabs 60 mm cage rotation mount for Ø2″ optics, LCRM2A/M	1	£124.50	£124.50	https://www.thorlabs.com/thorproduct.cfm?partnumber=LCRM2A/M	Metal
2-6	Thorlabs 60 mm cage plate with SM2 threads, and 0.5″ thickness (one SM2RR Retaining Ring included), LCP08/M	1	£52.15	£52.15	https://www.thorlabs.com/thorproduct.cfm?partnumber=LCP08/M	Metal
2-7	Thorlabs aspheric condenser lens, ACL5040U	1	£42.05	£42.05	https://www.thorlabs.com/thorpro523 nm.duct.cfm?partnumber=ACL5040U	Glass
2-8	Thorlabs extra-thick SM2 threaded retaining ring, SM2RRC	1	£11.70	£11.70	https://www.thorlabs.com/thorproduct.cfm?partnumber=SM2RRC	Metal
**2**-**4. QP mask**						
2-4-1(3D print)	QPM_holder_cover(PLA-CF)	1	∼£0.033/g (material)	<£0.50 (material)	https://uk.store.bambulab.com/products/pla-cf?variant=40577634271292	Polymer
2-4-2	Quadrant diffusers.Cut from A4 Tracing Paper (https://www.ryman.co.uk)	One piece	<£10.00 per pad	<£10.00	Most stationers, https://www.ryman.co.uk/stationery/	Non-specific
2-4-3	Quadrant polarisers.Cut from Edmund 100x100mm high contrast linear polarizing film (XP42-40)	1	£34.85	£34.85	https://www.edmundoptics.com/p/100-x-100mm-linear-polarizing-film-xp42-40/52988/	Polymer film
2-4-4(3D print)	QPM_holder_base(PLA-CF)	1	∼£0.033/g (material)	<£0.50 (material)	https://uk.store.bambulab.com/products/pla-cf?variant=40577634271292	Polymer
2-4-5(3D print)	QPM_quadrant_cutmodel(PLA-CF)	1	∼£0.033/g (material)	<£1.00 (material)	https://uk.store.bambulab.com/products/pla-cf?variant=40577634271292	Polymer
2-4-5 (CNC)	QPM_quadrant_cutmodel(Stainless steel)	1	∼£0.60/kg (material)	<£0.10 (material)	https://www.greengatemetals.co.uk/scrapmetal/Stainless+Steel.html	Metal

**3. Calibration masks**						
3-1(3D print)	CaliMask_source(PLA-CF)	1	∼£0.033/g (material)	<£0.50 (material)	https://uk.store.bambulab.com/products/pla-cf?variant=40577634271292	Polymer
3-2-1(3D print)	CaliMask_pupil(PLA-CF)	1	∼£0.033/g (material)	<£0.50 (material)	https://uk.store.bambulab.com/products/pla-cf?variant=40577634271292	Polymer
3-2-2(CNC)	CaliMask_pupil_adapter(Brass)	1	∼£3.60/kg (material)	<£0.50 (material)	https://www.greengatemetals.co.uk/scrapmetal/Brass.html	Metal

**4. PolCam**						
4-1	FLIR USB 3.1 Blackfly® S monochromatic polarized camera (PolCam), BFS-U3-51S5P-C	1	£1,921.00	£1,921.00	https://www.teledynevisionsolutions.com/products/blackfly-s-usb3/?model=BFS-U3-51S5P-C&vertical=machine%20vision&segment=iis	Non-specific
4-2	FLIR USB 3.1, 3 m, Type-A to Micro-B (locking) cable	1	£19.70	£19.70	https://www.flir.co.uk/products/usb-3.1-locking-cable/	Non-specific

## 6 Build instructions

In this section, the designators in Table 2 are used in figures as labels and in text as references inside parentheses.

### 6.1 Fabricate QP mask

6.1.a. 3D print, or CNC as indicated in Table 1, these three parts: QPM_holder_cover (***2-4-1***), QPM_holder_base (***2-4-4***), and QPM_quadrant_cutmodel (***2-4-5***).

QPM_holder_cover (***2-4-1***) and QPM_holder_base (***2-4-4***) are designed to push-fit with each other so that the quadrants in between them can be held tightly in place. However, this might be impacted by the specific materials and printers used for 3D printing. In this case, the outer diameter of QPM_holder_cover (***2-4-1***) may require small adjustments to achieve the push-fit.

6.1.b. Cut four quadrant diffusers (***2-4-2***) and four quadrant polarisers (***2-4-3***) from a piece of tracing paper and the linear polarising film, respectively, by placing QPM_quadrant_cutmodel (***2-4-5***) at different positions and orientations with respect to the paper or film to cut and cutting along its edges.

**Fig. 2:**
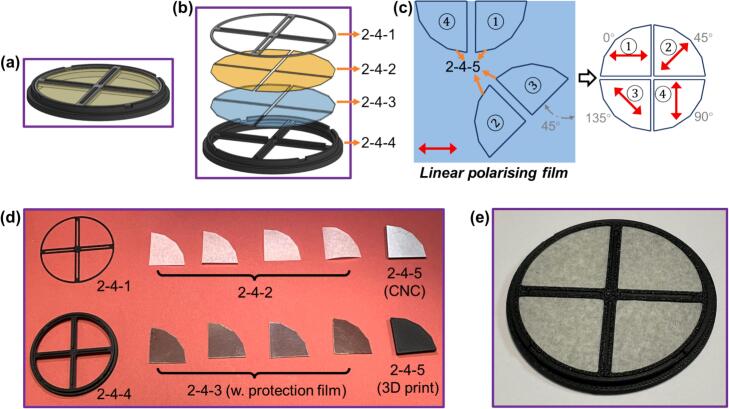
(a) The CAD and (b) the exploded view of QP mask (***2-4***); (c) Cut quadrant polarisers from a linear polarising film utilizing QPM_quadrant_cutmodel (***2-4-5***); (d-e) Photos of (d) all parts used during QP mask fabrication and (e) the fabricated QP mask (***2-4***).

Fig. 2(c) demonstrates one potential set of placements of QPM_quadrant_cutmodel (***2-4-5***) with respect to the linear polarising film, from which one can obtain four quadrant polarisers with the desired fast axis orientations.

6.1.c. With all parts needed to fabricate the QP mask (***2-4***) ready to hand, as shown in Fig. 2(d), peel off any protection film on the polarisers and assemble all parts except for the auxiliary part QPM_quadrant_cutmodel (***2-4-5***) according to Fig. 2(b). The final fabricated QP mask (***2-4***) should look like Fig. 2(e) from a top view.

### 6.2 Set up *openFrame*-based microscope for transillumination brightfield imaging

**Fig. 3:**
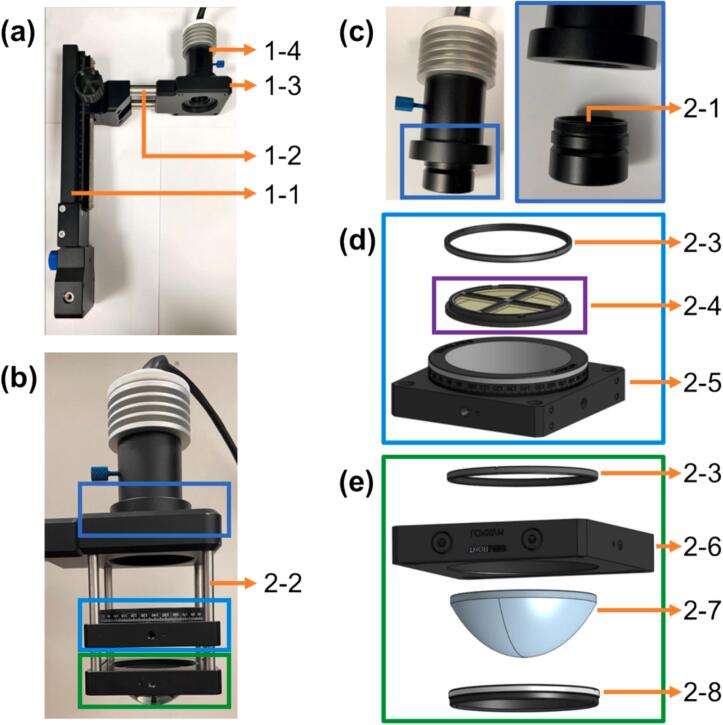
(a-b) Photos of openFrame transillumination arm for (a) brightfield imaging and (b) *pDPC* microscopy; (c) mounting of bandpass filter (***2-1***) in front of white monoLED (***1-4***); (d) exploded view of QP mask (***2-4***) mounted in the rotation stage (***2-5***); (e) exploded view of condenser lens (***2-7***) mounted in 60  mm cage plate 2***-***6.

Assemble the *openFrame*-based microscope stand, including the transillumination pillar and components for brightfield imaging (***1***), as shown in Fig. 3(a).

### 6.3 Set up PolCam

6.3.a. Mount the PolCam (***4-1***) onto one of the available camera ports (see Fig. 1) and connect the PolCam to the PC, preferably via a USB 3.0 (or better) port using a Type-A to Micro-B USB 3.0 (or better) locking cable (***4-2***).

6.3.b. Setup PolCam (***4-1***) in *µManager* using the Spinnaker device adapter by following the instructions provided at [26].

6.3.c. Adjust the axial position of PolCam (***4-1***) to ensure that the camera sensor coincides with the image (focal) plane of the tube lens.

If the microscope has another camera which has been set up in correct focus, this step can be achieved, e.g., by using that other camera to image a test chart sample at the sample (objective focal) plane and then adjusting the axial position of the PolCam (***4-1***) until a sharp image of the test chart is also formed on the PolCam (***4-1***).

Alternatively, the PolCam can be set to image a distant (no less than ∼ hundreds of meters) object through the tube lens, or a collimated low power laser beam may be directed to the tube lens to determine its focal plane on the PolCam (***4-1***) image side.

For convenience, adjust the rotation of PolCam (***4-1***) to align the edges of the camera sensor with the sample stage axes.

### 6.4 Set up the MM2_pDPC plug-in in *µManager*

Follow the installation and set-up documentation of the MM2_pDPC plug-in [23]. This plug-in will be utilized in Step 6.6.

### 6.5 Assemble the *pDPC* cage system

The *pDPC* cage system mainly consists of the QP mask (***2-4***) and the condenser lens (***2-7***), as well as their supporting optomechanical mounts. The distance between the QP mask (***2-4***) and the condenser lens (***2-7***) should be set up so that the quadrant diffusers (***2-4-2***) in the QP mask (***2-4***) coincide with the BFP of the condenser lens (***2-7***).

This can be achieved by the following steps:

6.5.a. Mount the condenser lens (***2-7***) in the 60 mm cage plate 2***-***6, as shown in Fig. 3(e).

6.5.b. Mount the QP mask (***2-4***) in the rotation mount (***2-5***), as shown in Fig. 3(d).

6.5.c. Mount the cage plate 2***-***6 and the rotation mount (***2-5***) in a 60  mm cage system constructed using four cage rods (***2-2***).

6.5.d. While fixing the 60 mm cage plate 2***-***6 near one end of the cage rods (***2-2***), slide the rotation mount (***2-5***) along the cage rods (***2-2***) until the quadrant diffusers (***2-4-2***) coincide with the condenser lens (***2-7***) BFP, and then fix the rotation mount (***2-5***) on the cage rods (***2-2***) at this position.

The BFP of the condenser (***2-7***) can be identified either by imaging objects far away or directing a collimated low power laser beam to the condenser lens and looking for the focus.

### 6.6 Set up the *pDPC* cage system on the *openFrame* transillumination arm

6.6.a. Mount the single-band bandpass filter (***2-1***) in front of MonoLED (***1-4***), as shown in Fig. 3(c). This is to create the quasi-monochromatic illumination required for *pDPC*. Ensure that the filter bandpass matches that of any other dichroic beamsplitters and emission filters in the light path between the sample and the PolCam.

6.6.b. Mount the *pDPC* cage system (***2***) onto the *openFrame* transillumination arm (***1***) by screwing one end of each of the four cage rods (***2-2***) into the four tapped holes at the bottom of the MonoLED adapter plate 1***-***3.

To maintain the distance between the QP mask (***2-4***) and the condenser (***2-7***), only one cage rod (***2-2***) should be loosened at a time during this mounting process. After mounting, the transillumination arm of the *openFrame* microscope should look like Fig. 3(b).

6.6.c. The height between the *pDPC* transillumination module (***2***) and the sample plane of the microscope does not have to exactly match the focal length of the condenser, given that light from each point source on the condenser BFP can be approximated to plane waves after collimation by the condenser lens. However, the closer this height is to the condenser focal length, the better the uniformity of illumination within the FOV will be.

In practice, this height can be adjusted by moving the whole transillumination arm up and down along the pillar (***1-1***), until images formed on all polarisation sub-image channels of the PolCam (***4-1***) are uniform across the FOV. To view polarisation sub-image channels of the PolCam in real-time, use the “***LiveSplit***” functionality in the MM2_pDPC plug-in (See [23] for details).

6.6.d. Align the orientation of QP mask (***2-4***) to that of the PolCam (***4-1***). The critical alignment is to ensure that the edges of the quadrant boundaries, i.e., the cross, at the middle of QPM_holder_base (***2-4-4***) are parallel or perpendicular to the sides of PolCam (***4-1***) sensor.

**Fig. 4:**
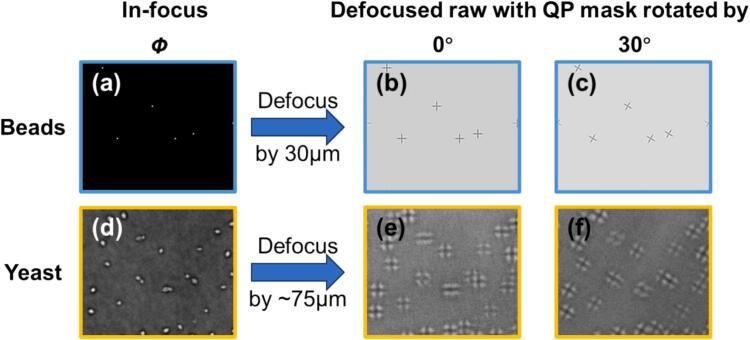
Effects of QP mask rotation on PolCam raw images of a slightly defocused sample: (a) ground-truth phase image of simulated beads sample; (b-c) simulated PolCam raw images of (a) defocused by 30 µm formed when QP mask is rotated at (b) 0 and (c) 30 degrees with respect to the PolCam sensor; (For details of simulation see Supplementary Information) (d) experimentally reconstructed phase image of yeast sample; (e-f) experimental PolCam raw images of (d) defocused by ∼75 µm formed when the QP mask is rotated at (e) 0 and (f) 30 degrees with respect to the PolCam sensor.

This can be adjusted either by directly imaging the BFP of the condenser lens onto the PolCam (***4-1***) by placing a Bertrand lens in the optical path (if available), or by imaging a slightly defocused sample (such as a bead sample), whose raw PolCam images vary with the rotation of QP mask (***2-4***) with respect to the PolCam (***4-1***) sensor as shown in Fig. 4.

## 7 Operation instructions

***pDPC* software**

**Fig. 5:**
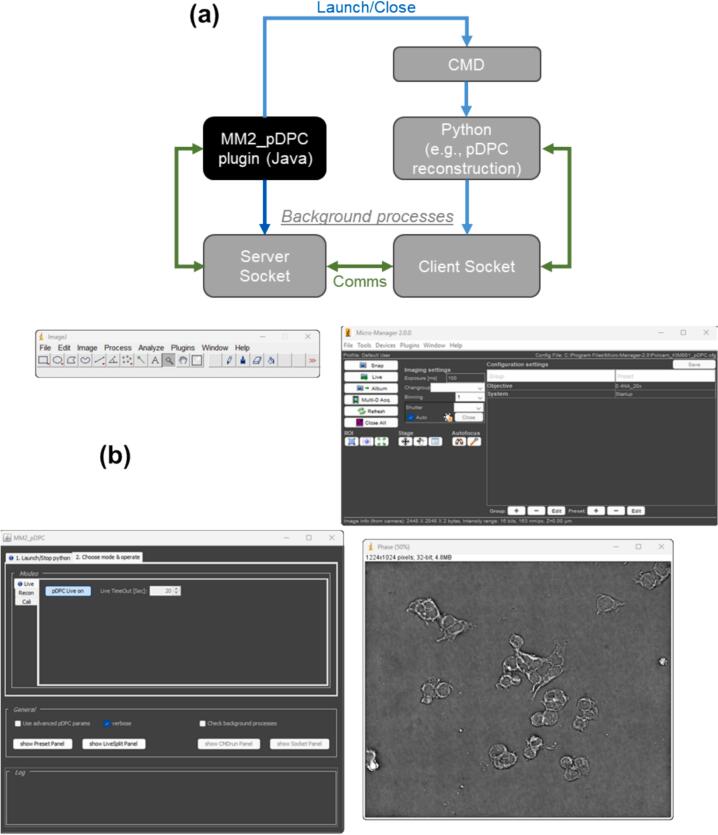
(a) Schematic of *pDPC* software configuration; (b) screenshot of *pDPC µManager* plug-in (MM2_pDPC).

The *µManager* plug-in (MM2_pDPC) providing *pDPC* image processing has a typical *µManager* GUI and can launch a background *python* program to perform complex image processing, such as *pDPC* phase image reconstruction (details see Supplementary Information). The general workflow of this plug-in and a snapshot of the GUI are shown in Fig. 5. Detailed information about the design of the software and an operator’s manual for this plug-in can be found at its public GitHub repository [23]. This *pDPC µManager* plug-in provides the functionality required during set-up and normal operation without requiring users to explicitly open/run any external windows or programs outside *µManager*.

The functionality of this plug-in includes the following options:

• ***Live***: on-the-fly reconstruction and display of phase images.
• ***Recon***: reconstructing and saving phase images from locally stored PolCam raw images.
• ***Cali***: analysing “calibration” data to produce the system response matrix for *pDPC* reconstruction.
• ***LiveSplit***: on-the-fly display of the four polarisation sub-image channels.

**Acquiring *pDPC* data**

If the *pDPC* system is already set up, routine operation typically entails acquiring a dark background image with no illumination. This is subtracted from subsequently acquired *pDPC* image data during the reconstruction process. If the set-up is known to be good, this is sufficient to obtain high quality phase images. However, when the *pDPC* system is set up for the first time, or if a misalignment is suspected to have happened, it is prudent to determine the system response, which is effectively the transmission matrix of the transillumination light from the quadrants of the QP mask to the pixels of the PolCam. If the system is suboptimal (e.g., due to misalignment of polarisation axes), this measured system response can be used instead of the (default) nominal system response (i.e., the transmission matrix assuming an ideal *pDPC* set-up with equal illumination from four quadrants in the QP mask, no crosstalk and equal sensitivity across polarisation channels) to correct the source functions used during phase reconstruction.

**Measuring the *pDPC* system response**

To maximise the (semi)quantitative phase imaging performance of a *pDPC* microscope, the system response, i.e., the transmission matrix for each polarisation-resolved illumination quadrant and the corresponding polarisation sub-image channel of the PolCam, can be determined by acquiring PolCam images with different illumination conditions but with no sample present. Essentially images are acquired with illumination being sequentially restricted to each of the quadrants in the QP mask. The resulting PolCam images can then be used to measure any crosstalk between the polarisation channels. When acquiring this data, it is recommended to acquire a continuous time series of images for each illumination condition to reduce the effect of noise. Dark background images (i.e., with no illumination) should also be acquired with the same PolCam exposure times used in order to facilitate dark current and camera offset subtraction.

**Fig. 6:**
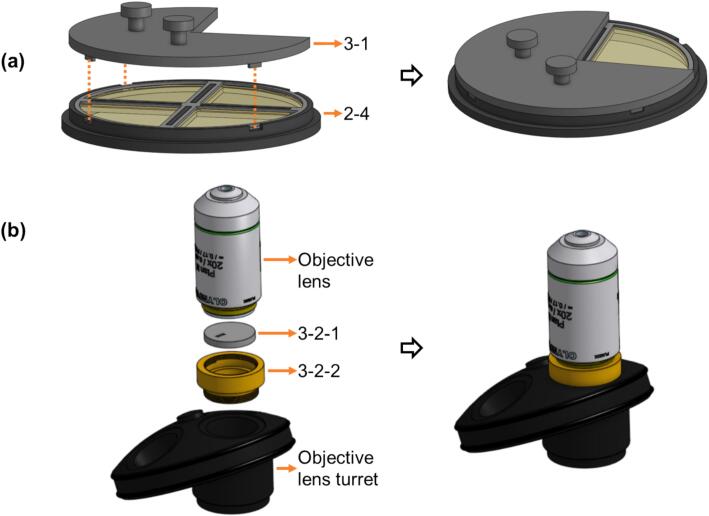
(a) Place CaliMask_source (***3-1***) on top of the QP mask (***2-4***) to block three quadrants. (b) Mount CaliMask_pupil (***3-2-1***) at the back of an objective by CaliMask_pupil_adapter (***3-2-2***).

First a set of light background images should be acquired when illuminating all the polarising quadrants in the source plane at the same time (i.e., normal *pDPC* illumination). Then, four sets of images should be acquired sequentially using a three-quadrant blocking mask (***3-1*** &/ ***3-2-1*** in Fig. 6) mounted at or near the source plane or the objective pupil plane such that transillumination light only passes through one quadrant light path at a time. The exposure time of the PolCam should be kept constant for these four sequential PolCam acquisitions. To confirm which quadrant is currently illuminated, the “***LiveSplit***” functionality of the MM2_pDPC plug-in can be used to indicate which polarisation sub-image channel has the highest detected intensity values.

There are two possible approaches to acquire the data needed to determine the *pDPC* system response:

**7.a. Determination of *pDPC* system response using a three-quadrant blocking mask at source plane**.

3D print the CaliMask_source (***3-1***) and place it on top of the QP mask (***2-4***). To only illuminate from one quadrant, rotate the CaliMask_source (***3-1***) so that its three pins fit into the holes on QPM_holder_base (***2-4-4***) as shown in Fig. 6(a).

**7.b. Determination of *pDPC* system response using blocking mask near objective lens pupil plane**

3D print the CaliMask_pupil (***3-2-1***) and CNC CaliMask_pupil_adapter (***3-2-2***). Mount them near the objective lens back focal plane as shown in Fig. 6(b). Rotate the CaliMask_pupil_adapter (***3-2-2***) to only detect light illuminated from one quadrant at a time.

These two approaches lead to comparable results. In most configurations of the *openFrame*-based microscope there is easy access to the source plane above the condenser lens. In other instruments, however, it may be more convenient to use the CaliMask_pupil (***3-2-1***) near the objective lens.

After acquiring the “calibration” image data, the “***Cali***” functionality of the MM2_pDPC plug-in is utilized to analyse the data and produce the overall *pDPC* system response in terms of the transmission matrix. Detailed steps explaining the use of this functionality can be found in the MM2_pDPC plug-in documentation at [23].

## 8 Validation and characterization

Following the steps in Section 6, *pDPC* was implemented on an *openFrame* microscope as shown in Fig. 1. To demonstrate the efficacy of measuring the system response, the system was “calibrated” using the CaliMask_source mask at the source plane and then using the CaliMask_pupil mask positioned near the objective lens pupil plane, following steps in Section 7.

**Fig. 7:**
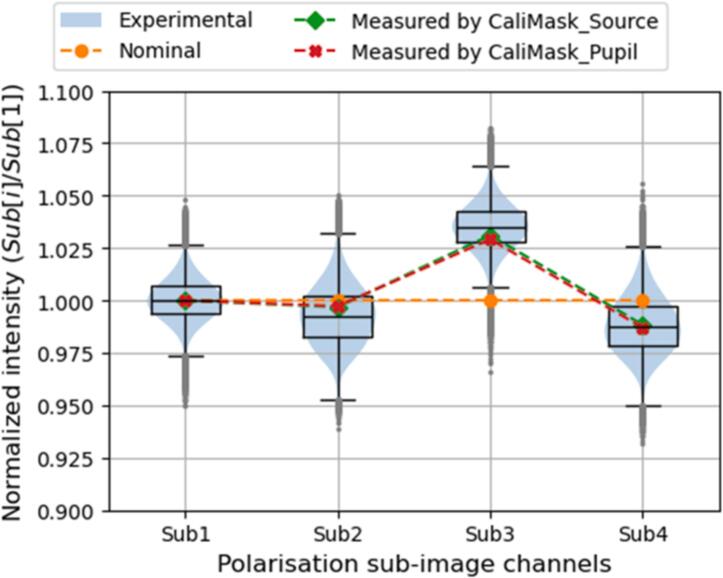
The theoretical and experimental normalized intensities at four polarisation sub-image channels.

Then, a no-sample light background image was acquired with all quadrants in the QP mask being illuminated. Four polarisation sub-image channels were extracted from the raw PolCam image and normalised by the median of the first polarisation sub-image channel. The distribution of normalized pixel intensities in four polarisation sub-image channels of the PolCam are shown in Fig. 7 where violin plots with blue shadows present the experimental pixel intensity histograms, black box plots highlight medians and quantiles, and grey dots indicate the outliers. The normalized intensities in polarisation sub-image channels expected for the nominal response matrix (i.e., the response matrix of an ideal *pDPC* set-up) are indicated as lines marked with orange circles. The normalized intensities calculated from the two measured response matrices obtained using the “calibration” masks at the source or near the objective lens pupil plane are indicated as lines marked with green diamonds and red crosses respectively.

It can be observed from Fig. 7 that the normalized intensities calculated from the two measured response matrices agree well with each other and are closer to the median of experimental normalised pixel intensity values for each polarisation sub-image channel than those calculated using the nominal response matrix. However, the deviations of the experimental normalized intensities and those calculated using the measured response matrices are mostly within 7.5 % of those expected for the nominal response matrix, suggesting that the system set-up is quite close to the ideal case, therefore requiring minimal correction during phase reconstruction.

**Fig. 8:**
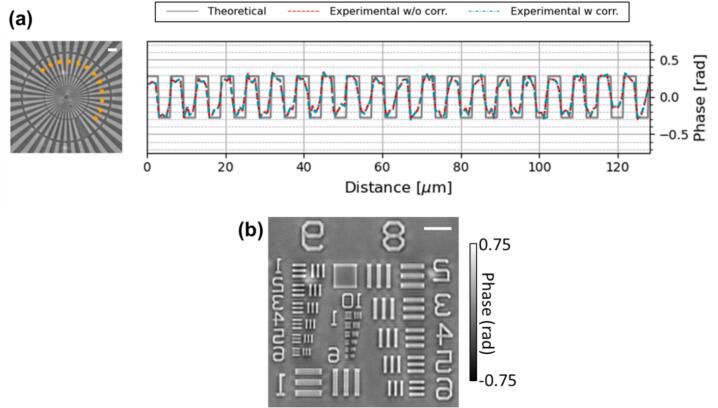
Phase images of a quantitative phase target. (a) Left: the phase image of a star chart acquired by a 10x 0.3NA objective and reconstructed using the nominal system response matrix; right: the expected phase profile along the orange dashed curve around the star chart (grey solid line) and the corresponding phase profiles from experimental measurements reconstructed with (cyan dashed line) and without (red dashed line) correction using the measured system response matrix. (b) The phase image of a USAF test chart acquired by a 20x 0.4NA objective and reconstructed using the nominal system response matrix, resolving up to Group 10 Element 1. (Scale bar: 10 µm).

To demonstrate the fidelity and resolution of the phase images provided by this *openFrame*-based *pDPC* microscope, Fig. 8 shows the results obtained by imaging a quantitative phase target (Benchmark Technologies, #991-2-1-8), which have become a *de facto* gold standard to evaluate QPI instruments. Fig. 8(a) presents the *pDPC* phase image of a star chart acquired by a 10x 0.3NA objective lens (Olympus UPLFLN10X) and demonstrates how the measured phase profiles (reconstructed with and without correction by the system response measured using CaliMask_pupil) for a circular path across the vanes of the star chart are approximately matched to the theoretical profile calculated from the manufacturer’s specifications. As discussed above and in [7], [27], in common with other differential phase contrast microscopy techniques, the assumption of the weak object and lack of low spatial frequency components mean that the periodic step phase profile is not fully recapitulated, although its scale and essential features are reproduced. Supplementary Fig. S5 shows how the limitations of differential phase contrast microscopy are manifest in *pDPC* phase images of a series of star charts with increasing feature height. Although the general morphology of the phase object is maintained, the deviation of the *pDPC* measurements from the theoretical profiles increases as the weak object assumption becomes less valid. We note the same trends are observed in [27]. Fig. 8(b) shows the *pDPC* phase image of a USAF test chart acquired by a 20x 0.4NA objective lens (Olympus PLN20X) that again illustrates the fidelity of the *pDPC* phase image and demonstrates that the spatial resolution is sufficient to resolve up to Group 10 Element 1 in the USAF test chart, which indicates a lateral resolution of ∼0.98 µm. For the 0.4NA objective lens used, the diffracted-limited spatial resolution is ∼0.79 µm at 628 nm. We note that the objective lens used is coverslip-corrected but there is no coverslip on the quantitative test target, and this may result in aberrations that slightly degrade the spatial resolution of the *pDPC* image.

**Fig. 9:**
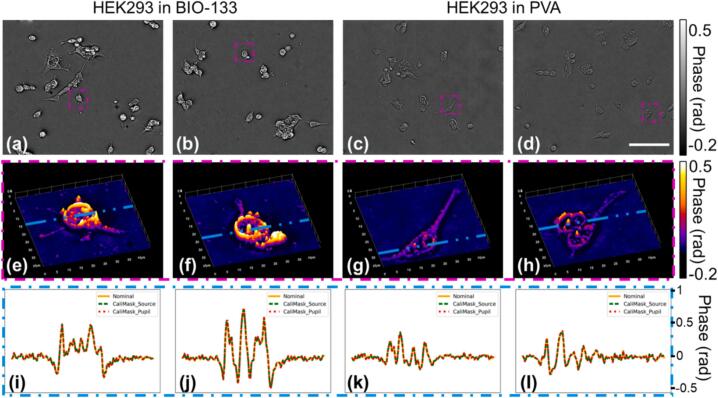
(a-d) Phase images of HEK293 cells fixed in (a-b) BIO-133 and (c-d) PVA media, respectively, reconstructed using the nominal system response matrix. (Scale bar: 100 µm) (e-h) Surface plots rendering phase values within purple boxes in (a-d), respectively. (i-l) Phase values along blue lines in (e-h) reconstructed by the nominal and measured system response matrices, respectively.

To illustrate the performance of this *openFrame*-based *pDPC* microscope on typical cell biology samples, Fig. 9 shows phase (OPD) images of HEK293 cells fixed and mounted in two different media, BIO-133 and poly (vinyl alcohol) Mowiol 4–88 (PVA), which were imaged using a 20x 0.4NA objective (Olympus PLN20X). The phase images were then reconstructed using both nominal and measured *pDPC* system response matrices. Fig. 9(a-d) present the phase images reconstructed by the nominal response matrix. The contrast of HEK293 cells in phase images differs between those embedded in BIO-133 and those in PVA. This can be explained by the different refractive indexes of these two types of mounting media. BIO-133 has a refractive index of ∼1.33 [28], while the PVA used in this experiment was formulated following [29] and has refractive index of ∼1.52. The line profiles of Fig. 9(i-l) shows only minor differences between phase images reconstructed with and without correction by the measured response matrices, confirming that the *pDPC* illumination and PolCam responses in this set-up present minimal crosstalk.

## CRediT authorship contribution statement

**Huihui Liu:** Writing – review & editing, Writing – original draft, Visualization, Software, Methodology, Conceptualization. **Sunil Kumar:** Writing – review & editing, Software, Methodology. **Edwin Garcia:** Writing – review & editing, Validation, Resources. **William Flanagan:** Writing – review & editing, Methodology. **Jonathan Lightley:** Writing – review & editing, Software, Methodology. **Christopher Dunsby:** Writing – review & editing, Methodology. **Paul M.W. French:** Writing – review & editing, Writing – original draft, Supervision, Conceptualization.

## Declaration of competing interest

The authors declare that they have no known competing financial interests or personal relationships that could have appeared to influence the work reported in this paper.
