# Supplementary material for: Open-source implementation of polarisation-resolved single-shot differential phase contrast microscopy (pDPC) on a modular openFrame-based microscope
Source: HardwareX. 2024 Dec 27;21:e00622. doi: 10.1016/j.ohx.2024.e00622 (PMC11773044; doi:10.1016/j.ohx.2024.e00622)
Supplement: Supplementary Data 1 [file mmc1.docx]

**Supplementary Information**

**The conventions below are used throughout this supplementary document.**

- $\boldsymbol{r}$ and $\boldsymbol{u}$ denote the 2D coordinates in the sample spatial and Fourier plane, respectively.
- $\tilde{f}(\boldsymbol{u}):={FT}_{2D} \left\{ f(\boldsymbol{r}) \right\}$ denotes the 2D Fourier transform (FT) of the function $f(\boldsymbol{r})$.

***pDPC* phase reconstruction**

**In our previous paper [**^[[1]](#endnote-1)^**] presenting the *pDPC* technique, we used the differential phase contrast software shared by Tian and Waller [**^[[2]](#endnote-2)^**,**^[[3]](#endnote-3)^**] by applying arithmetic operations on four polarisation sub-image channels (deinterleaved from a single-shot PolCam raw image) to synthesise two pairs of images required by their software inputs, which would have been obtained with each half aperture of the condenser back focal plane along the horizontal and vertical axes sequentially blocked. However, the MM2_pDPC plug-in reported here calculates phase images directly from four polarisation sub-image channels, albeit using essentially the same mathematics as in [2,3]. Figure S1 demonstrates how the phase image of a sample is reconstructed from a single-shot PolCam raw image in MM2_pDPC plug-in using the nominal system response matrix (i.e., assuming ideal *pDPC* system set-up).**


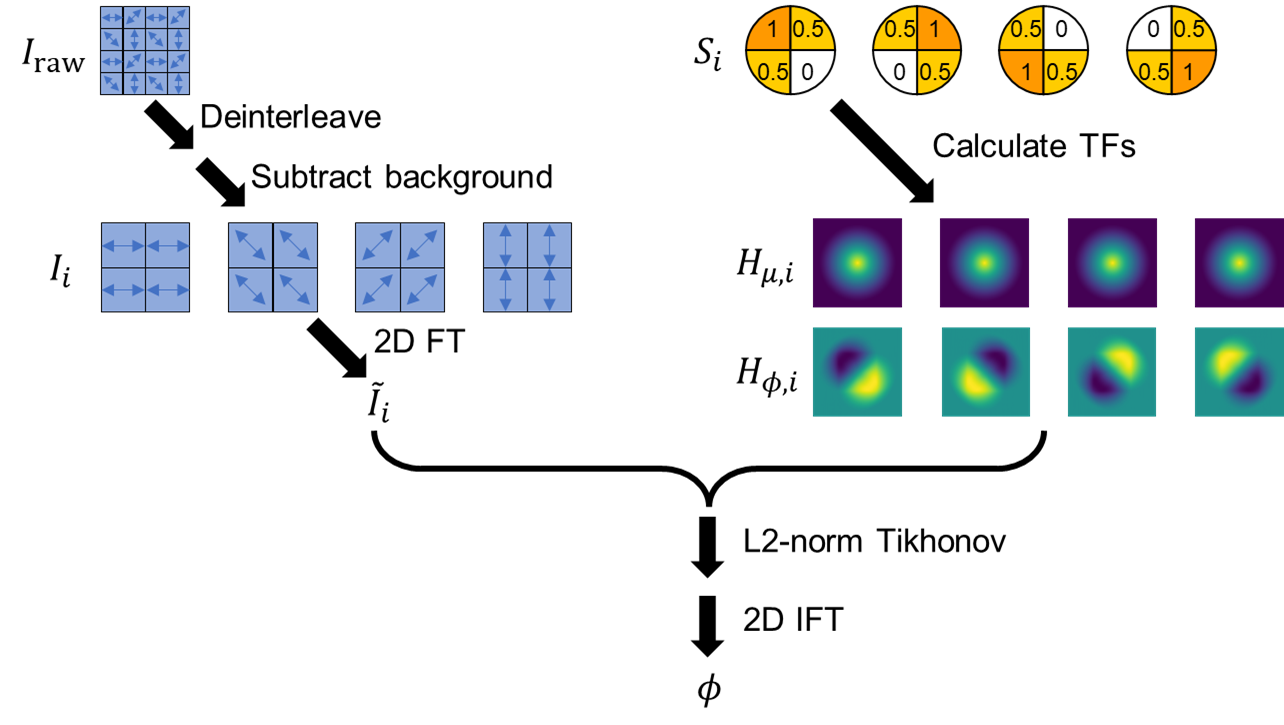


**Supplementary Figure S****1. Schematic of phase reconstruction process in the MM2_pDPC plug-in using the nominal system response matrix. (FT: Fourier transform; IFT: inverse Fourier transform; TF: transfer function)**

**Basically, u**nder the weak object assumption (WOA), the optical transmission function $o$ of a sample can be approximated as in Eq.(1).

|  | $o\left( \boldsymbol{r} \right)=e^{\mu\left( \boldsymbol{r} \right)+j\phi(\boldsymbol{r})}\underset{\to}{\mathrm{WOA}} 1+\mu\left( \boldsymbol{r} \right)+j\phi(\boldsymbol{r})$ | (1) |
| --- | --- | --- |

**where** $\phi$ and $\mu$ are the phase and amplitude parts of the sample transmission, respectively.

**After deinterleaving** the single-shot PolCam raw image $I_{\mathrm{raw}}$ into four sub-images $I_{i}^{raw}$ $(i=1,2,3,4)$ and subtracting the backgrounds $I_{i}^{background} (i=1,2,3,4)$ from the sub-images according to

|  | $I_{i}= \frac{I_{i}^{raw}}{I_{i}^{background}}-1$ | (2) |
| --- | --- | --- |

**the background-subtracted four polarisation sub-image channels** $I_{i}$ $(i=1,2,3,4)$ can be expressed in the Fourier domain using the vector form as in Eq.(3). Note that the background images can be determined from *pDPC* image acquisitions with no sample in place or they can be calculated by uniform filtering the sub-images $I_{i}^{raw}$ with a large kernel (usually at half image size) [2,3].

|  | $\tilde{\boldsymbol{I}}(\boldsymbol{u})=A(\boldsymbol{u})\times\tilde{\boldsymbol{o}}(\boldsymbol{u})$ | (3) |
| --- | --- | --- |

where

|  | $\tilde{\boldsymbol{I}}(\boldsymbol{u})=\left[ \begin{matrix} \tilde{I_{1}}(\boldsymbol{u}) \\ \tilde{I_{2}}(\boldsymbol{u}) \\ \begin{aligned} \tilde{I_{3}}(\boldsymbol{u}) \\ \tilde{I_{4}}(\boldsymbol{u}) \end{aligned} \end{matrix} \right], \tilde{\boldsymbol{o}}(\boldsymbol{u})=\left[ \begin{matrix} \tilde{\mu}(\boldsymbol{u}) \\ j\tilde{\phi}(\boldsymbol{u}) \end{matrix} \right], A(\boldsymbol{u})=\left[ \begin{matrix} H_{\mu,1}(\boldsymbol{u}) \\ H_{\mu,2}(\boldsymbol{u}) \\ \begin{aligned} H_{\mu,3}(\boldsymbol{u}) \\ H_{\mu,4}(\boldsymbol{u}) \end{aligned} \end{matrix}\begin{matrix} H_{\phi,1}(\boldsymbol{u}) \\ H_{\phi,2}(\boldsymbol{u}) \\ \begin{aligned} H_{\phi,3}(\boldsymbol{u}) \\ H_{\phi,4}(\boldsymbol{u}) \end{aligned} \end{matrix} \right]$ |  |
| --- | --- | --- |

Using this, the phase $\phi$ of the sample can be calculated via L2-norm Tikhonov regularization and then inverse Fourier transform (IFT) as in Eq.(4)-(5).

|  | $\tilde{\boldsymbol{o}}\boldsymbol{=}\left( A^{T}A\boldsymbol{+\rho}\mathbb{I} \right)^{-1}A^{T}\tilde{\boldsymbol{I}}$ | (4) |
| --- | --- | --- |
|  | $\Rightarrow\phi(\boldsymbol{r})=\mathrm{IFT}_{2D}\left\{ \tilde{\phi}(\boldsymbol{u}) \right\}$ | (5) |

where $\boldsymbol{\rho}$ is the vector of regularization parameters and $\mathbb{I}$ denotes the identity matrix.

$H_{\phi,i}\left( \boldsymbol{u} \right)$ and $H_{\mu,i}\left( \boldsymbol{u} \right)$ denote the system phase and amplitude transfer functions (TFs) corresponding to the $i$-th **polarisation sub-image channel and** can be calculated as in Eq.(6)-(7), respectively.

|  | $H_{\phi,i}\left( \boldsymbol{u} \right)=\frac{TCC_{i}\left( \boldsymbol{u} \right)-TCC_{i}^{*}\left( -\boldsymbol{u} \right)}{TCC_{i}(\boldsymbol{0})}$ | (6) |
| --- | --- | --- |
|  | $H_{\mu,i}\left( \boldsymbol{u} \right)= \frac{TCC_{i}\left( \boldsymbol{u} \right)+TCC_{i}^{*}\left( -\boldsymbol{u} \right)}{TCC_{i}(\boldsymbol{0})}$ | (7) |

where ${TCC}_{i}$ denotes the transmission cross coefficient calculated as in Eq.(8).

|  | $TCC_{i}\left( \boldsymbol{u} \right)=\int S_{i}\left( \boldsymbol{u}^{'} \right)\cdot P\left( \boldsymbol{u}^{'}+\boldsymbol{u} \right)\cdot P^{*}\left( \boldsymbol{u}^{'} \right) d\boldsymbol{u}'$ | (8) |
| --- | --- | --- |

$P\left( \boldsymbol{u} \right)$ denotes the pupil function of the system, in this case, a disk function whose aperture is defined by the objective NA. $S_{i}\left( \boldsymbol{u} \right)$ denotes the source function corresponding to each **polarisation sub-image channel, which essentially reflects their transmission efficiencies for each illumination quadrant polariser as well as the relative intensity ratios of illumination from each quadrant.** Illustrations of $S_{i}\left( \boldsymbol{u} \right)$ for an ideal *pDPC* set-up are presented in Figure S**1**. However, in practical experiments the values in each quadrant of $S_{i}\left( \boldsymbol{u} \right)$ can vary and may be corrected by setting the values according to the “calibrated” system response matrix.

**Simulation of raw PolCam images with rotated QP mask in Figure 4**

The PolCam raw images demonstrated in Figure 4(b-c) were generated by forward simulating a pure phase sample with $\mu(r)=0$ and $\phi(r)$ as shown in Figure 4(a) imaged under a *pDPC* microscope setup with a 20x 0.4 NA objective and 628 nm 0.55 NA Köhler illumination. During simulation, all system response matrices were set to nominal values assuming ideal system set-ups and the background was set as the same for all four polarisation sub-image for simplicity.

To simulate the forward imaging process of *pDPC* for a sample with a known transmission function $o(\boldsymbol{r})$, four **polarisation sub-image channels** $I_{i}(\boldsymbol{r})$ are first calculated respectively by Eq.(3) and then interleaved into a single-shot PolCam raw image $I_{\mathrm{raw}}(\boldsymbol{r})$. The effect of a rotated QP mask and a defocused sample is introduced in the former step by modifying the source and pupil functions, which will then change the subsequent system amplitude and phase TFs, as well as the final images.

Note that the rotation of the QP mask changes the range of illumination angles of light from each quadrant, as well as the response factors at polarisation sub-image channels, both of which are demonstrated in Figure S2 and have been considered during calculation of Figure 4(b-c).


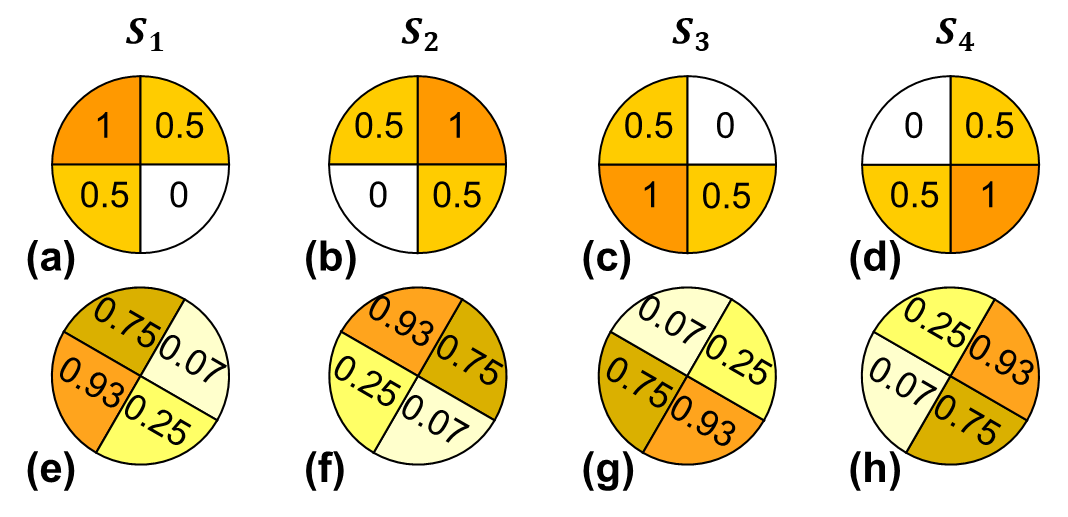


**Supplementary Figure S****2.** (a-d) show the ideal polarisation channel source functions and their relative channel transmission efficiencies and (e-h) show the transmission efficiencies when the QP mask was rotated from 0 to 30 degrees orientation relative to the detection polarisation.

To incorporate the defocus effects, the pupil function $P\left( \boldsymbol{u} \right)$ in Eq.(8) is substituted by $P\left( \boldsymbol{u},z \right)$ in Eq.(9) during calculation.

|  | $P\left( \boldsymbol{u},z \right)=P\left( \boldsymbol{u} \right)\cdot e^{j2\pi\cdot\eta\cdot z}, \eta^{2}+\left\vert\boldsymbol{u} \right\vert^{2}=\lambda^{-2}$ | (9) |
| --- | --- | --- |

where $z$ is the defocus distance of the sample from the objective focal plane.

**Combined *pDPC* and fluorescence imaging**

Supplementary Figure S3 illustrates the ability to capture both *pDPC* and fluorescence image data of the same field of view. This is being explored for label-free cell segmentation and tracking for high content assays.


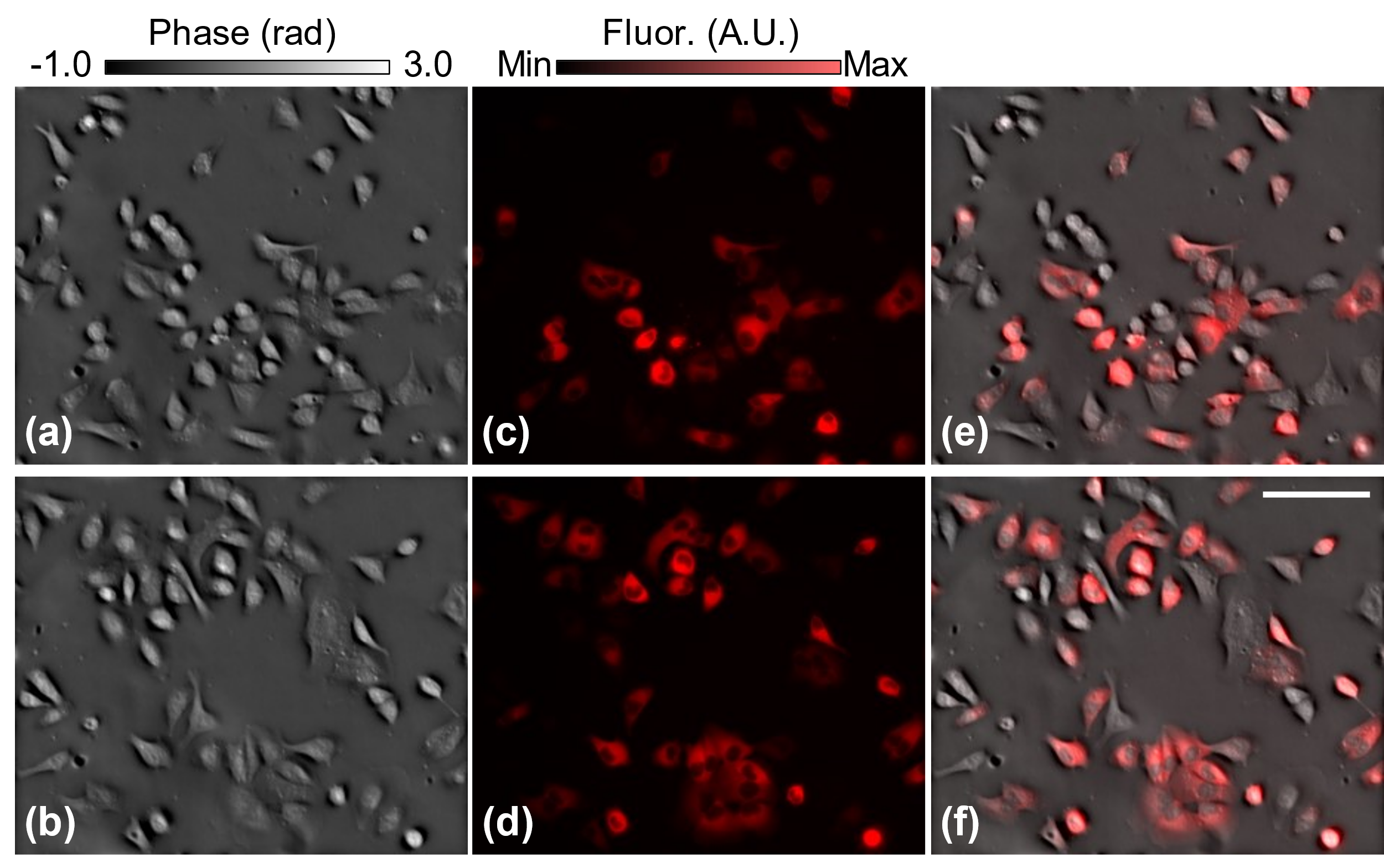


**Supplementary Figure S****3**. (a-b) phase images, (c-d) fluorescence images, and (e-f) their merged images of live DU145 cells labelled with mTurquoise2FP in PBS medium acquired using a 20x water immersion objective on an *openFrame*-based microscope configured for both *pDPC* and epifluorescence. (Scale bar: 100 µm)

**Effect of correcting system response matrix**

Supplementary Figure S4 shows *pDPC* images of (M. smegmatis) mycobacteria, illustrating that the resolution achieved is close to the diffraction-limited value (noting that M. smegmatis is expected to have a diameter ~300-500 nm [^[[4]](#endnote-4)^]); Figure S4(b-g) illustrate the impact of applying the “calibration” procedure to correct the *pDPC* system response matrix for polarisation crosstalk. While the correction makes negligible difference to the base experimental *pDPC* system (indicating that it is well-aligned), the impact is more significant after a dielectric coated beamsplitter was inserted into the optical path that increased the crosstalk between the polarisation channels.

**
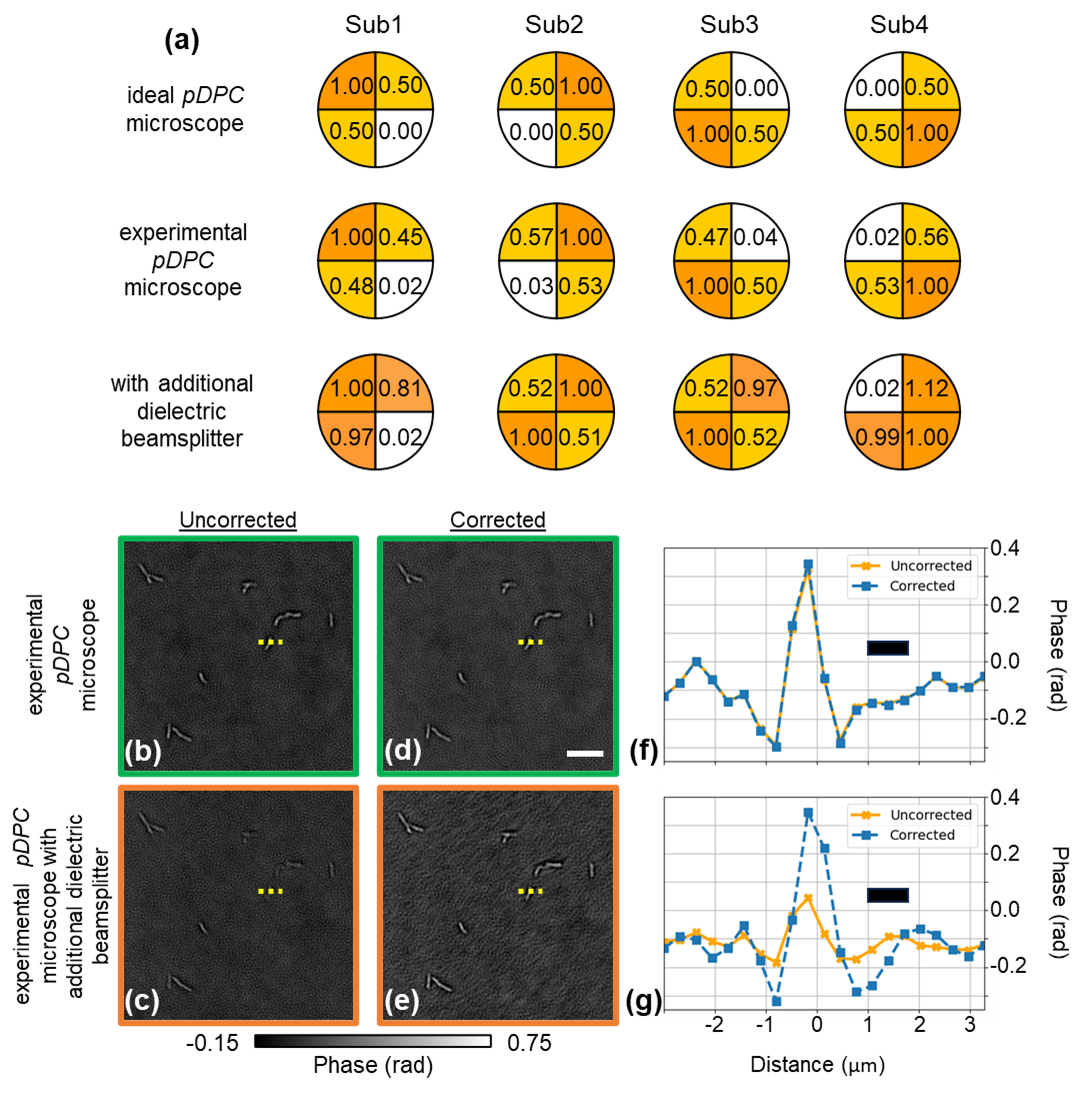
**

**Supplementary Figure S****4**. **(a)** transmission efficiencies to the PolCam sub-images for each polarisation channel (Sub1-Sub4) for light from the four illumination quadrant polarisers in the ideal case (top row), the experimental *pDPC* microscope (middle row) and the same experimental microscope with an additional dielectric beamsplitter inserted in the infinity space (bottom row).
**(b-e)** *pDPC* phase images of (M. smegmatis) mycobacteria fixed in BIO-133 media, acquired using a 20x 0.4NA objective (Olympus PLN20X) lens (b,d), and also with an additional dielectric coated beamsplitter inserted in the infinity space (c,e), showing reconstructions with (d,e) and without (b,c) correction by the system response matrices corr. to the transmission ratios shown in (a). (Scale bar: 10 µm)
**(f-g)** line profiles corresponding to yellow dashed lines in (b,d) and (c,e), respectively. Scale bars indicate the diffraction limited resolution for the 0.4 NA objective lens at a wavelength of 628 nm.

***pDPC* images of star charts with increasing feature height**

Supplementary Figure S5 illustrates how the performance of the *pDPC* system is impacted by increasing deviation from the weak object assumption as the feature height of the sample phase profile is increased. From top to bottom in Figure S5, the shape of the *pDPC* phase profile seems to be well maintained as the feature height of the star chart increases but the measured phase values become noticeably different from the theoretical values (calculated using the manufacturer’s specifications). This trend is consistent with previous observation in the literature on differential phase contrast microscopy, e.g., [^[[5]](#endnote-5)^].


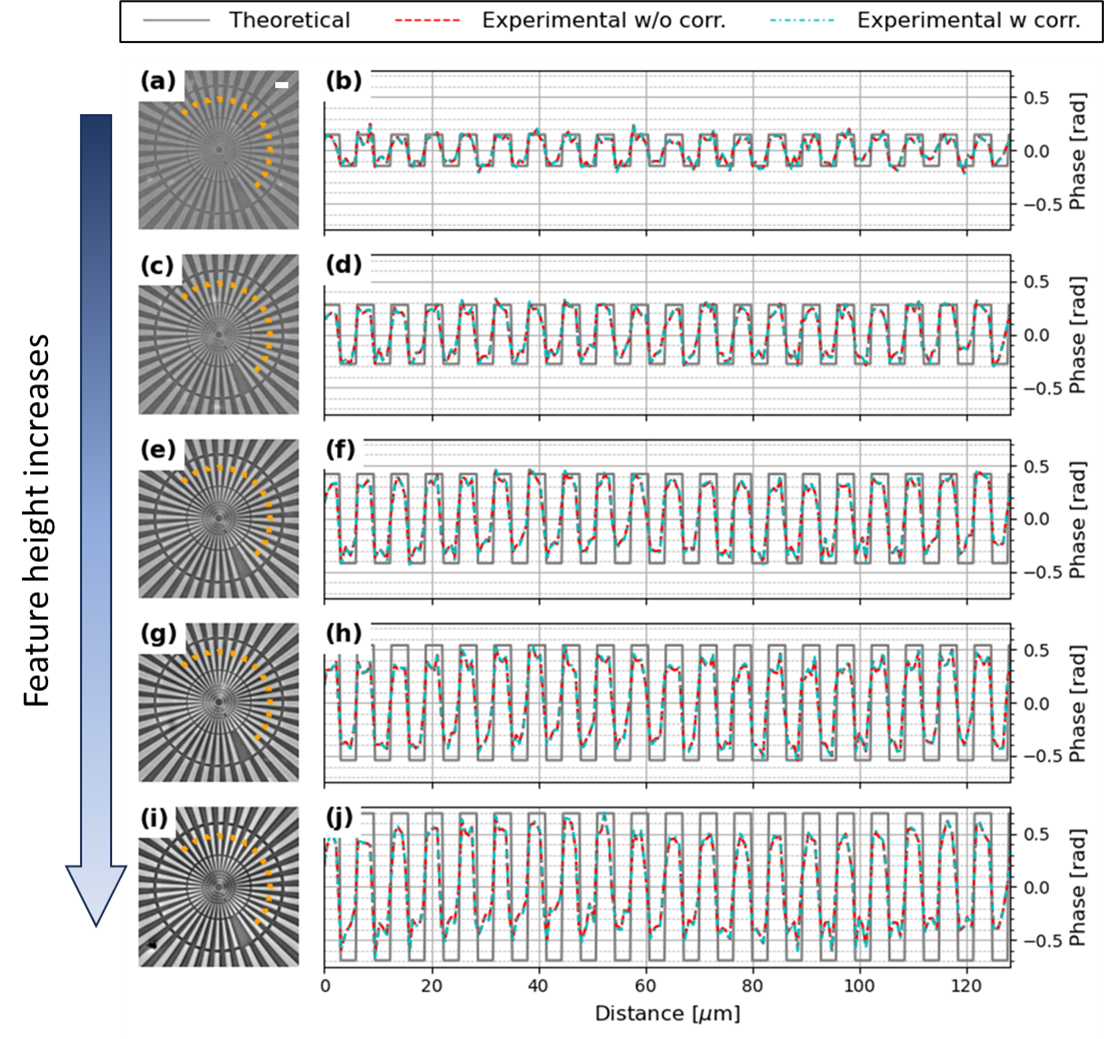


**Supplementary Figure S****5**. Left: phase images (a,c,e,g,i) of star charts with increasing feature height on a quantitative phase target (Benchmark Technologies, #991-2-1-8), acquired by a 10x 0.3 NA objective lens (Olympus UPLFLN10X) and reconstructed using the nominal system response matrix. (Scale bar: 10 µm) Right: phase profiles (b,d,f,h,j) along the dashed orange curves in (a,c,e,g,i) with theoretical (grey solid lines) and experimentally measured phase profiles reconstructed with (cyan dashed lines) and without (red dashed lines) correction using the measured system response matrix.

**Effect of camera signal level**

Supplementary Figure S6 illustrates the improvement in performance of the *pDPC* system with increasing signal levels of images of mycobacteria acquired on the PolCam. The “noise” in the phase imaging can be parameterised by the standard deviation (SD) of the calculated phase in regions where there is no sample, e.g. [^[[6]](#endnote-6)^]. A segmentation mask of mycobacteria was generated by *ImageJ* *MaxEntropy* thresholding the phase image acquired at the maximum illumination level to differentiate between the background and regions where the sample (mycobacteria) are present. At each illumination intensity level, the SD of background phase values were calculated over the FOV for each of 16 frames acquired sequentially and the median of these SD values is plotted as a function of camera signal (green triangles). To indicate the variation of the uncertainty of the calculated phase values as a function of camera signal, at each illumination intensity level, the SD of sample phase values for each pixel were calculated across the 16 frames acquired sequentially and the median of these SD values is plotted (blue crosses).


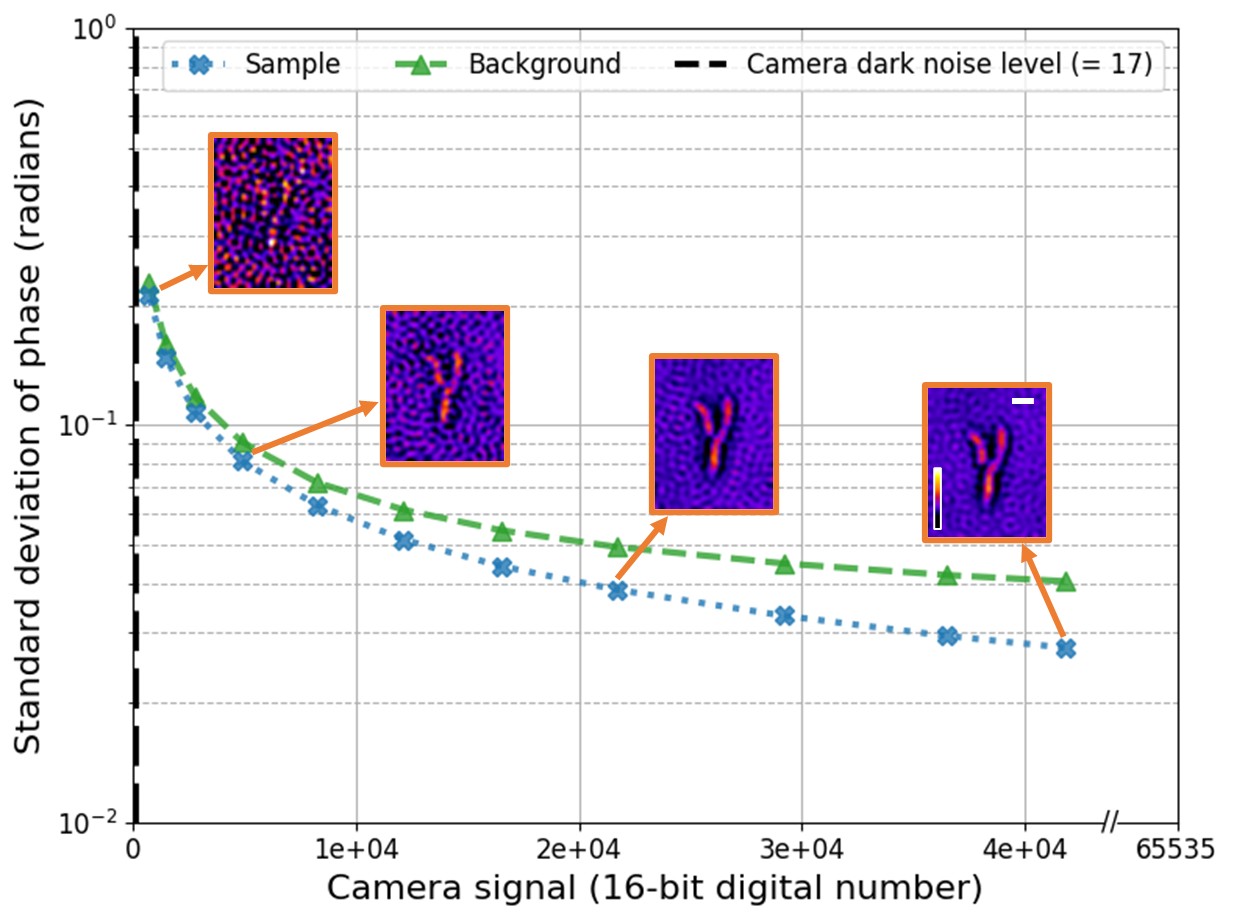


**Supplementary Figure S****6**. Plots of standard deviations of background phase “noise” (▲) and uncertainty on calculated sample phase (**x**) as a function of camera signal level measured by *pDPC* imaging of M. smegmatis mycobacteria fixed in BIO-133 using a 20x 0.4 NA objective (Olympus PLN20X) lens, acquiring 16 frames consecutively at 11 different illumination light intensity levels. Inset images are of the same mycobacteria imaged at different illumination intensities. (Scale bar: 2 µm) Vertical dashed black line is the camera dark noise level.

**References**

1. R. Kalita et al., "Single-shot phase contrast microscopy using polarisation-resolved differential phase contrast," J Biophotonics, p. e202100144, Aug 13 2021, doi: 10.1002/jbio.202100144 [↑](#endnote-ref-1)
2. “DPC”, <https://github.com/Waller-Lab/DPC>**. (Accessed 10/06/24)** [↑](#endnote-ref-2)
3. L. Tian and L. Waller, "Quantitative differential phase contrast imaging in an LED array microscope," Opt Express, vol. 23, no. 9, pp. 11394-403, May 4 2015, doi: 10.1364/OE.23.011394 [↑](#endnote-ref-3)
4. G.M. Cook, M. Berney, S. Gebhard, M. Heinemann, R.A. Cox, O. Danilchanka, M. Niederweis, Physiology of mycobacteria., Adv. Microb. Physiol. 55 (2009) 81-182,318-319. https://doi.org/10.1016/S0065-2911(09)05502-7. [↑](#endnote-ref-4)
5. M. Chen, Z.F. Phillips, L. Waller, Quantitative differential phase contrast (DPC) microscopy with computational aberration correction, Opt. Express 26 (2018) 32888–32899. https://doi.org/10.1364/OE.26.032888. [↑](#endnote-ref-5)
6. H. Lu, J. Chung, X. Ou, and C. Yang, "Quantitative phase imaging and complex field reconstruction by pupil modulation differential phase contrast," Opt Express, vol. 24, no. 22, pp. 25345-25361, Oct 31 2016, doi: 10.1364/OE.24.025345. [↑](#endnote-ref-6)
